# MRI response rate after short-course radiotherapy on rectal cancer in the elderly comorbid patient: results from a retrospective cohort study

**DOI:** 10.1186/s13014-020-01500-y

**Published:** 2020-03-02

**Authors:** T. Koëter, S. G. C. van Elderen, G. F. A. J. B. van Tilborg, J. H. W. de Wilt, D. K. Wasowicz, T. Rozema, D. D. E. Zimmerman

**Affiliations:** 1 Department of Surgery, Elisabeth-TweeSteden Hospital Tilburg, Tilburg, The Netherlands; 2 Department of Surgery, Radboud University Medical Centre, Geert Grooteplein Zuid 10, 6525 GA Nijmegen, The Netherlands; 3 Department of Radiology, Elisabeth-TweeSteden Hospital Tilburg, Tilburg, The Netherlands; 4 Department of Radiotherapy, Verbeeten Instituut Tilburg, Tilburg, The Netherlands

**Keywords:** Rectal cancer, Radiotherapy, Magnetic resonance imaging, Chemoradiotherapy, MRI, Short-course radiotherapy, mrTRG, Restaging

## Abstract

**Background:**

The aim of the present study was to evaluate MRI response rate and clinical outcome of short-course radiotherapy (SCRT) on rectal cancer as an alternative to chemoradiotherapy in patients where downstaging is indicated.

**Methods:**

A retrospective analysis was performed of a patient cohort with rectal carcinoma (cT1-4cN0-2 cM0–1) from a large teaching hospital receiving restaging MRI, deferred surgery or no surgery after SCRT between 2011 and 2017. Patients who received chemotherapy during the interval between SCRT and restaging MRI were excluded.

The primary outcome measure was the magnetic resonance tumor regression grade (mrTRG) at restaging MRI after SCRT followed by a long interval. Secondary, pathological tumor stage, complete resection rate and 1-year overall survival were assessed.

**Results:**

A total of 47 patients (M:F = 27:20, median age 80 (range 53–88) years), were included. In 33 patients MRI was performed for response assessment 10 weeks after SCRT. A moderate or good response (mrTRG≤3) was observed in 24 of 33 patients (73%). While most patients (85%; *n* = 28) showed cT3 or cT4 stage on baseline MRI, a ypT3 or ypT4 stage was found in only 20 patients (61%) after SCRT (*p* <  0.01). A complete radiologic response (mrTRG 1) was seen in 4 patients (12%). Clinical N+ stage was diagnosed in *n* = 23 (70%) before SCRT compared to *n* = 8 (30%) post-treatment (*p* = 0.03).

After SCRT, 39 patients underwent deferred surgery (after a median of 14 weeks after start of SCRT) and a resection with complete margins was achieved in 35 (90%) patients. One-year overall survival after surgery was 82%. Complete pathological response was found in 2 patients (5%).

**Conclusions:**

The use of SCRT followed by a long interval to restaging showed a moderate to good response in 73% and therefore can be considered as an alternative to chemoradiotherapy in elderly comorbid patients.

## Introduction

Colorectal cancer is currently the second most common cancer in the Netherlands, with an incidence of approximately 5000 rectal cancer patients per year. Over the past decades, there have been significant developments in the treatment of rectal cancer.

Local recurrence rates have significantly decreased due to the introduction of TME (total mesorectal excision) surgery combined with short-course radiotherapy (SCRT) [1, 2]. In the Netherlands, this combined treatment has been the cornerstone in management of resectable rectal cancer in the past decade and leading to excellent results [3]. According to the Dutch Colorectal Cancer Guidelines, patients diagnosed with intermediate risk rectal cancer (T1-T3 with positive lymph node staging (N1) or T3 N0 with > 5 mm extramural invasion and uninvolved MRF) are treated with neoadjuvant SCRT and are planned for immediate surgery. In case of a threatened mesorectal fascia (cT3/4MRF+) and/or multiple positive lymph nodes (cN2), patients are usually treated with long course CRT followed by restaging and TME resection in case of sufficient downstaging or individualized treatment in persistent LARC [4].

Tumor response rate at restaging MRI and predictors of a favorable outcome after chemoradiotherapy (CRT) has been studied before [5–7]. However, a clinical problem arises when patients with a LARC are unfit to undergo chemoradiotherapy (CRT) due to extensive comorbid conditions, a problem well-described in a recent review [8]. Comorbid conditions (i.e. cardiovascular comorbidity) and high age in patients with advanced tumors are the main reason to choose a SCRT regimen followed by deferred resection [9]. A scheme of SCRT (consisting of 25 Gy (Gy), administered in 5 fractions during 1 week), followed by a delayed MRI assessment of tumor downstaging can be a treatment option [10, 11]. In the Stockholm 3 trial, one treatment arm consisted of patients randomized for SCRT and a long interval to surgery (4–8 weeks), resulting in an ypT0N0 (pathological complete response (pCR)) rate of 11.8% [12]. Besides downstaging and even the possibility of a pCR after a prolonged interval, SCRT followed by long interval has several advantages. Firstly, SCRT is well tolerated and radiation-induced toxicity is only seen in about 6% of patients during the resting period before surgery. Secondly, the risk of surgical complications is significantly reduced by delaying surgery compared to standard immediate surgery [10]. Therefore, it may even be justified to defer surgery after SCRT in selected cases.

Few data are available on the effects of SCRT on tumor size, lymph node status and resection margins when surgery is deferred. In 2016, a prospective cohort consisting 18 patients reported complete symptom resolution in almost 40% of the patients in a palliative setting while Cummings et al. described a similar effect in 20 patients [13, 14]. Bujko et al. published an interim subgroup analyses of 30 patients who underwent SCRT followed by long interval prior to reassessment and reported a clinical complete response (cCR) rate of 20% [15]. Thus, there seems to be a lack of published data specifically on MRI response after SCRT and a prolonged interval. The present retrospective study was undertaken to investigate the MRI response rate of rectal cancer after SCRT followed by a long interval. Besides MRI response, oncological outcomes after deferred surgery in a selected more comorbid population were evaluated. We hypothesized that SCRT followed by a long interval can be a viable alternative to CRT in a selected, more comorbid population.

## Methods

Included patients met the following criteria: patients diagnosed with rectal cancer (cT1-4cN0-2 cM0–1) who received SCRT and underwent deferred surgery, patients who received SCRT and underwent restaging MRI or patient who underwent SCRT that did not undergo surgery due to be considered unfit for CRT, due to high age in combination with frailty or due to multiple distant metastasis. Patients were treated between 2011 and 2017 and all patients were discussed within a multidisciplinary oncological team (MDT). All patients underwent MR imaging (MRI) in order to determine a treatment strategy. Short-course radiation therapy consisted of 25 Gy administered in 5 fractions during 1 week. Information on the patients’ characteristics, such as sex and date of birth, American Society of Anaesthesiologists (ASA) score, comorbidity, tumor characteristics, stage (clinical and pathological TNM classification), histology, details from surgical treatment and perioperative complications were registered. An involved circumferential resection margin (CRM) was defined as the presence of tumor cells within 1 mm of the lateral surface of the mesorectal fascia (MRF). Exclusion criteria were immediate surgery after RT, recurrent rectal cancer or patients who received chemotherapy during the interval between SCRT and surgery.

Primary outcome was defined as radiological response rate (Magnetic Resonance Tumor Regression Grade(mrTRG)) on restaging MRI after SCRT followed by a long interval. As a secondary outcome, 1-year overall survival, pathological staging and complete resection rate were evaluated.

### Radiological review

Staging MRI and re-staging MRI after radiotherapy treatment were independently reviewed by two dedicated abdominal radiologists with respectively 5 and 15 years of experience in rectal cancer MRI reporting. If inconsistent findings were reported, consensus was reached through discussion.MRIs were reviewed for: tumor (T) stage, distance from anorectal junction, mesorectal fascia involvement, lymph node status, magnetic resonance tumor regression grade (mrTRG). MrTRG consists of five grades: see Table 1 [16].

**Table 1:** Classification system for mrTRG

Description	Grade
Complete regression (absence of tumour signal and barely visible treatment related scar)	mrTRG 1
Good regression (predominant low signal intensity fibrosis with no obvious areas of intermediate signal intensity)	mrTRG 2
Moderate regression (low signal intensity fibrosis predominates but there are obvious areas of intermediate signal intensity)	mrTRG 3
Slight regression (little areas of low signal intensity fibrosis or mucin but mostly tumor)	mrTRG 4
No regression (intermediate signal intensity, same appearances as original tumor)	mrTRG 5

*mrTRG* Magnetic resonance tumour regression grade

The recently updated European Guideline for Magnetic Resonance Imaging from the European Society of Gastrointestinal Abdominal Radiology (ESGAR) was used in the reviewing process [17]*.*

### Statistical analyses

Descriptive statistics were expressed as median and standard deviation (SD)) for continuous variables. Differences between groups were calculated by using the Mann-Whitney U test for continuous variables. The Pearson χ2 test or the Fisher’s exact tests, if appropriate, were used for categorical variables. Statistical significance was considered at *p* <  0.05. Statistical analyses were performed using SPSS software version 23 (IBM, Armonk, New York, USA).

## Results

A total of 47 patients were included: baseline characteristics are shown in Table 2.

**Table 2:** Baseline characteristics

	5 × 5 Gy radiotherapy (*n* = 47)
< 75 years	15 (31.9)
≥ 75 years	32 (68.1)
Sex (male)	27 (57.4)
Clinical characteristics	
ASA	
I	3 (6.4)
II	14 (29.8)
III	26 (55.3)
IV	4 (8.5)
Number of comorbid conditions	
None	2 (4.3)
One	16 (34.0)
Two or more	29 (61.7)
Tumor distance from anal verge in cm (median (range))	6 (0–15)
Stage 4 disease at diagnosis	7 (14.9)
Follow up in months (median (range))	29.9 (8.5–94.1)

Data are n (%) if not otherwise specified

*ASA* American Society of Anaesthesiologists

The majority (*n* = 32, 68.1%) of patients were 75 years or older and were diagnosed with multiple comorbidities (61.7% had 2 or more), corresponding ASA classification is shown in Table 2. Median distance from the anorectal junction was 6.0 cm (range 0–15 cm). Of the 47 included patients, 7 patients (14.9%) had synchronous metastases.

Median follow up in months after start of radiotherapy was 29.9 months (range 8.5–94.1).

### Primary outcome (tumor response on MRI)

Of the included 47 patients, 33 patients were staged using an MRI pre- and post-radiotherapy. Clinical tumor stage, cN stage, MRF involvement and mrTRG are shown in Table 3 based on pre- and post-RT MRI. Seventy-three percent of patients showed at least a moderate to good response to SCRT (mrTRG ≤3; *n* = 24 of 33). In 4 patients (12.1%) a radiological complete response was suspected. After surgery, in three out of four patients with a suspected radiological complete response residual tumor was found (2 ypT2; 1 ypT1 tumor). Median time between the start of radiotherapy and restaging MRI was 10 weeks (range 6–45).

**Table 3:** MRI outcomes

	MRI pre-treatment (*n* = 33)	MRI post-treatment (*n* = 33)		*p*-value
cT stage				< 0.001
T0	0	4 (12.1)		
T1	0	0		
T2	5 (15.2)	9 (27.3)		
T3A/B	11 (33.3)	8 (24.2)		
T3C/D	7 (21.2)	4 (12.1)		
T4A	6 (18.2)	5 (15.2)		
T4B	4 (12.1)	3 (9.1)		
cN stage				< 0.001
N0	10 (30.3)	25 (75.8)		
N1	12 (36.4)	5 (15.2)		
N2	11 (33.3)	3 (9.1)		
MRF involvement				0.12
Yes	17 (51.5)	13 (39.4)		
No	16 (48.5)	20 (60.6)		
mrTRG				
Grade 1		4 (12.1)		
Grade 2		5 (15.2)		
Grade 3		15 (45.5)		
Grade 4		8 (24.2)		
Grade 5		1 (3.0)		

Data are n (%) if not otherwise specified

*cT* Clinical tumor stage, *cN* Clinical nodal stage, *MRF* Mesorectal fascia, *mrTRG* Magnetic resonance tumour regression grade

MRI of patients post-RT treatment showed downstaging (cT stage) compared to pre-RT treatment MRI, this difference was statistically significant (*p* <  0.001). Based on the pre-RT MRI, 28 patients (51.5%) were staged with a locally advanced (T3C/D, T4A/B) tumor compared to 12 patients (36.4%) post-RT, this difference was statistically significant (*p* <  0.001). Clinical N0 stage was diagnosed in 10 patients (30.3%) pre-RT treatment compared to 25 (75.8%) patients post-RT treatment (*p* = 0.03). There was no statistically significant difference in mesorectal fascia involvement (17 patients MRF + before SCRT vs 13 patients after SCRT (*p* = 0.12)).

In Table 4, the correlation between cT-stage at restaging MRI and ypT-stage of the patients who received a restaging MRI and subsequent deferred surgery is shown.

**Table 4:** Correlation between T-stage at restaging MRI and pathologic T-stage of patients who underwent subsequent deferred surgery

	Number of patients	Pathologic stage				
Restaging MRI		ypT0	ypT1	ypT2	ypT3	ypT4
cT0	3	0	1	2	0	0
cT1	0	–	–	–	–	–
cT2	8	1	2	2	3	0
cT3	9	1	0	2	6	0
cT4	8	0	0	1	5	2

### Outcomes after surgery

Thirty-nine out of 47 included patients underwent deferred surgery after SCRT, 35 patients underwent transabdominal laparoscopic rectal resection according to the TME principle and 4 patients underwent a local excision using TransAnal Minimally Invasive Surgery (TAMIS) procedure [18, 19]. Pathological tumor and lymph node stage are shown in Table 5. One-year overall survival rate was 82.1%. Complete pathological response was found in 2 patients (5.1%). A resection with clear margins was achieved in 32 TME cases (91.4%). Median time between start of radiotherapy and surgery was 14 (range 7–26) weeks.

**Table 5:** Outcomes after surgery

	Surgery^a^ (*n* = 39)
ypT stage	
Complete response	2 (5.1)
T1	4 (10.3)
T2	9 (23.1)
T3	21 (53.8)
T4	3 (7.7)
ypN stage^b^	
N0	22 (56.4)
N1	8 (20.5)
N2	5 (12.8)
Harvested lymph nodes (median (range))^b^	15 (8–31)
Tumor positive lymph nodes (median (range))	0 (0–12)
Complete resection (CRM-)^b^	32 (91.4)
Local recurrence	3 (7.9%)
30-day mortality	7.7%
Stage 4 disease at time of surgery	5 (12.8)
1 year overall survival	32 (82.1)

Data are n (%) if not otherwise specified

^a^ 4 patients underwent local excision though TAMIS procedure

*ypT* Pathological tumor stage, *ypN* Pathological nodal stage, *CRM* Circumferential resection margin

^b^only patients who underwent Total Mesorectal Excision (TME)

A total of 3 local recurrences were diagnosed during follow up; one was diagnosed through endoscopy and confirmed by histology, in 2 patients a local recurrence was suspected on Computed Tomography (CT) of the pelvis. Median time of recurrence after surgery was 5.9 months (range 4.1–24.9).

### Outcomes without surgery

Eight out of 47 patients never underwent surgery. Reasons to refrain from surgery were the presence or development of multiple distant metastasis (*n* = 3), extensive comorbidity combined with high age (*n* = 3), one patient refused surgery and further treatment and one patient died 6 weeks after radiotherapy treatment of massive hemorrhage from the tumor. Six out of 8 patients died at the time of analysis, median overall survival was 19 months (range 1–35 months) after radiotherapy treatment. Follow up was limited due to the palliative setting in which these patients were treated, 2 patients were reirradiated successfully because of an increase of pain following tumor growth. One patient was admitted to the hospital for palliative sedation due to untreatable pain at home.

## Discussion

The primary aim of this retrospective cohort study was to evaluate the radiological response of SCRT treatment followed by long interval on patients diagnosed with rectal cancer. Despite being widely used all over the world, there are surprisingly few data available concerning this treatment regimen. Comparing pre- and post-treatment MRI, the present study shows a moderate to good response rate (mrTRG ≤3) in 72.8% of patients treated with SCRT. A significantly higher rate of negative lymph node staging was found in post-RT treatment MRI’s when compared to pre-RT treatment MRI’s.

The mrTRG grading was designed by Patel et al. [5]. These authors described 5 different magnitudes of response (as depicted in Table 1). Sclafani et al. evaluated the mrTRG in patients treated with neoadjuvant CRT for locally advanced rectal cancer [20]. In their study, they found a mrTRG ≤3 in 143 of 191 (74.9%) patients. This seems in accordance with our findings. However, when comparing patients with a favorable mrTRG 1–2, the group of Sclafani found a larger proportion of responders than the present study does. Sclafani et al. found a percentage of 45.5% in the CRT group whereas the current study only found 27.2%. This finding is not surprising, as the superior effect of CRT over SCRT is well documented, however the similarity in the mrTRG< 3 group between the study of Sclafani and the present study is noteworthy.

In our results, 4 (12%) patients had a radiological complete response (mrTRG 1) following SCRT treatment based on the post-treatment MRI. However, histopathological findings showed residual tumor in 3 of them (2 ypT2; 1 ypT1). Sclafani et al. compared mrTRG with pathological TRG (pTRG) and found that the agreement between these two modalities are low and stated that mrTRG cannot be used as a surrogate of pTRG. Especially when an organ preserving treatment is considered, the addition of digital rectal examination and endoscopy are key [21]. At this moment however, mrTRG has shown to be the most reliable of current methods for assessing response prior to surgery [22].

In a large nationwide study from Rombouts et al., a complete pathological response was found in 9.3% of patients after SCRT with long interval, compared to 5.1% in the present study [23]. Factors that can contribute to the difference in ypCR-rate are a more advanced tumor stage in our cohort (T3 and/or T4: 84.8% vs. 73.2% by Rombouts et al.) and a longer interval to surgery (14 weeks in our study vs. 8.1 weeks by Rombouts et al.). Nonetheless, the 5.1% complete response rate in the present study is in line with a trial from Latkauskas et al., who described a complete response rate of 4.4% [24].

Besides tumor response, lymph node status was assessed on MRI pre- and postradiotherapy treatment. Clinical N0 stage was more frequently diagnosed after radiotherapy treatment compared to MRI before treatment (75.8% vs. 30.3% (*p* = 0.03)). These results should be interpreted with caution, since the assessment of suspicious lymph nodes on MRI before and after neoadjuvant chemoradiation treatment has proven to be only moderately reliable [25–27]. Besides these limitations, MRI still is preferable for N-stage assessment because it allows the evaluation of the whole mesorectum.

Postoperative lymph node involvement was seen in 13 out of 35 cases (37.2%), while the remaining 22 cases were N0 (62.9%). The lymph node yield in our population who underwent TME surgery seems to be appropriate, with a median of 15 lymph nodes in the specimens and in the majority (80%) of patients at least 12 lymph nodes yielded [28].. A study from Garcia-Florez et al. investigated tumor and lymph node response to chemoradiation therapy in an locally advanced setting [29]. Their population was comparable to our group in means of tumor staging on MRI, but less co-morbid. After chemoradiotherapy, they found a negative lymph node involvement of 68.5% in the resected specimens, compared to 64.7% in our cohort. Therefore, we conclude that SCRT and long interval to surgery seems to induce an adequately down staging in lymph node stage.

Despite the small number, a percentage of 8.6% (3/35) incomplete resections (R1) is relatively high in our opinion. Involved circumferential resection margin (CRM) is an important risk factor for locoregional recurrence and nowadays one of the important aims of rectal cancer therapy is to avoid R1 resections [30]. Two of the three patients with involved margins where operated after extensive discussion in MDT’s on our hospital and an academic locally-advanced referral center and suffered from tumor perforation with pelvic sepsis and tenesmi with limited distant disease, respectively. In one patient the peritoneum covering the urinary bladder appeared to be involved, which was not to be seen on preoperative MRI.

Local recurrence rate was 7.9% after a median follow up of 29.9 months after surgery, in 1 out of 3 patients an involved CRM was found in the resected specimen. A Cochrane review from 2012 described local recurrence rates in locally advanced rectal cancer as a primary outcome parameter [31]. After CRT, a local recurrence rate of 9% was found compared to 13.4% after radiotherapy treatment alone [32]. Although most patients in our cohort were diagnosed with cT3/T4 tumors (84.8%), a lower local recurrence rate should be pursued. Nowadays, a further centralization of rectal cancer treatment, especially in a locally advanced setting, is effectuated.

The overall 1-year survival rate of 82.1% seems to be low compared to a 2-year survival rate of 82% from the Dutch TME trial and a 3-year survival rate of 86.7% from the COLOR II trial [2, 33]. However, the patients included in our cohort represent a frailer and comorbid population, based on higher age and ASA classification, with a substantial number of locally advanced tumors.

Of course, main limitations of this study are the relatively small number of included patients so one must be cautious extrapolating these date for general practice. Furthermore, the retrospective design has its obvious disadvantages.

## Conclusion

In conclusion, this is the first study evaluating radiological response of rectal cancer on short-course radiotherapy treatment. The use of short-course radiotherapy followed by a long interval seems to be an acceptable alternative to chemoradiotherapy in a selected population where, due to a comorbid or frail condition, an individualized treatment is warranted.

## Data Availability

The datasets generated and analysed during the current study are not publicly available due privacy reasons but are available from the corresponding author on reasonable request.

## Abbreviations

ASA: American society of anaesthesiologists
cCR: Clinical complete response
CRM: Circumferential resection margin
CRT: Chemo radio therapy
DRE: Digital rectal examination
Gy: Gray
LARC: Locally advanced rectal cancer
MDT: MultiDisciplinary oncological team
MRF: MesoRectal fascia
MRI: Magnetic resonance imaging
mrTRG: Magnetic resonance tumor regression grade
pCR: Pathological Complete Response
pTRG: Pathological tumor regression grade
RT: Radio therapy
SCRT: Short course radio therapy
TAMIS: TransAnal minimally invasive surgery
TME: Total mesorectal excision
